# Is Fibrin a Suitable Matrix for Small-Diameter Bioartificial Vascular Grafts? An In Vitro Short-Term Hemocompatibility Study

**DOI:** 10.3390/jfb17060303

**Published:** 2026-06-18

**Authors:** Clara Glomb, Patrick Stadler, Melanie Klingenberg, Michael Pflaum, Arjang Ruhparwar, Mathias Wilhelmi, Florian Helms

**Affiliations:** 1 Lower Saxony Center for Biomedical Engineering, Implant Research and Development (NIFE), Stadtfelddamm 34, 30625 Hannover, Germany; 2 Division for Cardiothoracic-, Transplantation- and Vascular Surgery, Hannover Medical School, Carl-Neuberg-Str. 1, 30625 Hannover, Germany; 3 Department of Vascular- and Endovascular Surgery, St. Bernward Hospital, 31134 Hildesheim, Germany

**Keywords:** vascular tissue engineering, Chandler Loop, vascular graft, regenerative medicine, hemocompatibility, fibrin matrix

## Abstract

**Background:** The generation of durable and hemocompatible small-diameter vascular grafts remains a major challenge in current vascular tissue engineering, as clinically available synthetic grafts are lacking hemocompatibility resulting in limited long-term patency. In recent years, fibrin has emerged as a promising scaffold material for various tissue engineering approaches due to its autologous nature, controllable fabrication, and mechanical properties. However, although pivotal for the translation into clinical application, systematic evaluation of hemocompatibility in fibrin-based small-caliber grafts is still missing. **Methods:** Here, the hemocompatibility of small-diameter fibrin-based grafts with and without heparin coating was compared to the current gold standard for prosthetic small-diameter vessel replacement in the form of heparin-coated ePTFE grafts using the Chandler Loop circulation model with human whole blood. Cell adhesion of thrombocytes, erythrocytes, and leucocytes was compared. Platelet activation, activation of the complement system, and plasmatic coagulation activity were assessed by ELISA analyses for P-Selectin, complement sC5b-9, and thrombin–antithrombin complex, respectively. Scanning electron microscopy (SEM) was performed to evaluate interactions and thrombocyte activation on the luminal graft surfaces. **Results:** The short-term hemocompatibility of the fibrin-based grafts with respect to the cell-count, activation of the coagulation pathways, and thrombocyte activation was comparable to the heparin-coated synthetic grafts even without heparin coating of the bioartificial grafts. **Conclusions:** The findings of this early-stage analysis support fibrin as a promising scaffold material for small-diameter vascular tissue engineering.

## 1. Introduction

Cardiovascular diseases are the number one cause of death worldwide [1]. With particular relevance, small-vessel diseases such as coronary artery disease or peripheral artery disease often require surgical implantation of vascular grafts in the form of bypass operations.

While good long-term results can be achieved with large-diameter synthetic grafts composed of expanded polytetrafluorethylene (ePTFE) or polyethylene terephthalate (Dacron^®^) [2,3], one of the major drawbacks of these fabrics is insufficient patency especially in small-diameter grafts (<6 mm in diameter) [4]. Several strategies have been developed in the past to overcome this limitation in synthetic small-diameter vascular prostheses. As one of the most common modifications, heparin coating has been established and is currently used to reduce thrombogenicity and increase patency rates in small-diameter synthetic grafts. However, with patency rates below 50% after 5 years [5], even heparin-coated grafts cannot be considered a viable option for small-diameter vessel replacement to date. The clinical alternative to synthetic vascular prosthesis is autotransplantation of native small-diameter blood vessels. This technique, however, is not infrequently complicated by limited availability and varying quality of autologous transplantable vascular grafts. Thus, currently available techniques for small-diameter vascular replacement surgery are highly limited with respect to small-diameter vessels resulting in a great clinical need for innovative vascular graft materials. When used as vessel replacements with small diameters, these must meet the highest requirements in terms of hemocompatibility in order to prevent premature prosthesis occlusion.

In recent years, vascular tissue engineering has emerged as a promising approach for the generation of bioactive vascular grafts that provide potential benefits compared to the currently available synthetic graft materials. For this, fibrin has been identified as a promising matrix material in various tissue engineering approaches [6]. Due to its availability from the peripheral blood [7] and the controllable polymerization process [8,9,10], fibrin is especially suitable for vascular tissue engineering applications [11].

With these properties, fibrin-based bioartificial vascular grafts are of interest especially for small-diameter vessel replacement. In previous works, we and others established a variety of fabrication techniques for small diameter fibrin-based vessels and proofed the applicability of fibrin as a matrix material for vascular tissue engineering in vitro and in vivo [8,9,12,13]. Particularly in small-diameter grafts, excellent hemocompatibility is of pivotal importance to avoid graft thrombosis and occlusion. While bioartificial fibrin-based grafts theoretically offer advantages over synthetic grafts in this area, hemocompatibility of fibrin-based small-diameter vascular grafts has not been investigated systematically to date.

Thus, we here provide an in vitro hemocompatibility analysis of fibrin-based grafts with a diameter of 5 mm in comparison to commercially available heparin-coated ePTFE-grafts currently used in clinical practice. Furthermore, potential effects of heparin-coating of fibrin-based vessels on the hemocompatibility were investigated. For this, the extracorporeal circulation system Chandler Loop^®^ was used, which allows in vitro hemocompatibility analysis under dynamic incubation using human blood [14,15,16].

## 2. Materials and Methods

### 2.1. Generation of Fibrin-Based Vascular Grafts and Sample-Preparation

**Figure 1:**
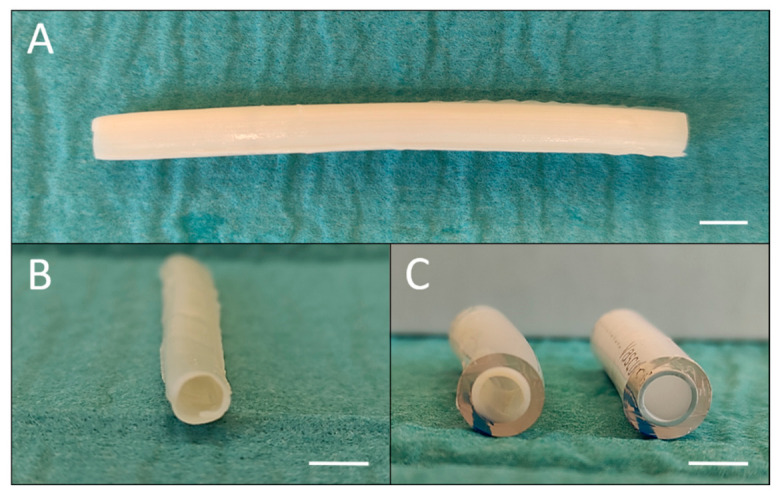
Macroscopic graft morphology. (**A**): abluminal top view of a small-diameter fibrin-based graft; (**B**): exemplary cross section of fibrin-based graft; (**C**): comparative cross section of specimen after installation into tube, left: fibrin graft, right: synthetic graft. Scale bar: 5 mm.

Fibrin-based vascular grafts were generated as previously described [8]. Briefly, cryoprecipitated fibrinogen was added to a solution of 100 µL × mL^−1^ Medium 199 10× (Sigma Aldrich, Steinheim, Germany), 1 µL × mL^−1^ Aprotinin (Bayer, Leverkusen, Germany) as well as titrated with 1N NaOH for pH-adaption resulting in a final fibrinogen concentration of 25 mg × mL^−1^. To initiate the polymerization process, a solution containing 0.5 U × µL^−1^ Thrombin and 0.06 U × µL^−1^ Factor XIII (CSL Behring, Marburg, Germany) supplemented with a 40 mM CaCl_2_ solution (CSL Behring, Marburg, Germany) was added to the fibrinogen solution to initiate polymerization. Subsequently, the mixture was filled into a cylindrical casting mold with an outer diameter of 5 mm, that was equipped with a central placeholder to generate the vessel lumen. This step was followed by a 15 min incubation period. After initial polymerization, an angioplasty dilatation catheter (Charger^TM^ 6 F, 5.0 mm × 200 mm; 135 cm, Boston scientific, Marlborough, MA, USA) was used to apply transluminal compression in order to compact the fibrin matrix and remove excess fluid for a total of 60 min. The resulting fibrin-based vascular grafts were 10 cm long with an outer diameter of 5 mm (Figure 1A,B).

Following this protocol, two fibrin-based vessels were fabricated for each test run. One of the fibrin grafts in each run was additionally treated with a heparin coating using a two-step approach: First, the fibrin graft was incubated in a solution containing 5 U × µL^−1^ thrombin replenished in phosphate-buffered saline (PBS) for one hour. Afterwards, the specimen was submerged in a solution containing 5 U × µL^−1^ Heparin for one hour. Luminal heparin-coating was verified using immunohistochemical analysis and immunofluorescence microscopy for an epitope 10E4 antibody (Ab Heparan Sulfate, clone F58-10E4, amsbio, Oxford, UK).

For the Chandler Loop setup, phthalate-free PVC-based tubes (noDOP^®^-tubes) were used. In order to properly and securely install the samples into the Chandler Loop System, all specimen were prepared in a separate tube segment with a length of 3 cm. To facilitate a firm inherence of the sample to the luminal surface of the tube segment, a thin layer of medical device adhesive (Loctite 4011^®^, Henkel Adhesives Technologies, Düsseldorf, Germany) was applied to the inside of the tube prior to the insertion of the graft. The fibrin vessel was mounted onto a deflated angioplasty catheter (POWERFLEX™ PRO 6F, Cordis Corporation, Miami, FL, USA) and inserted into the tube segment lumen. The catheter was then dilated until firm attachment of the fibrin grafts to the inner surface of the tube was achieved. Additionally, the construct was submerged shortly into a PBS solution to fully facilitate secure consolidation of the moisture-activated adhesive. After an incubation period of 15 min, the catheter was deflated and carefully removed. This resulted in tube segments with firmly inserted samples as shown in Figure 1C. For comparison to the current clinical gold standard for synthetic small-diameter grafts, an ePTFE-based heparin-coated graft (Vascugraft^®^ Flow, strait tube, 5 mm, B. Braun, Melsungen, Germany) was inserted into the tube of the Chandler Loop System in the same manner.

### 2.2. In Vitro Testing in the Chandler Loop Circulation System

**Figure 2:**
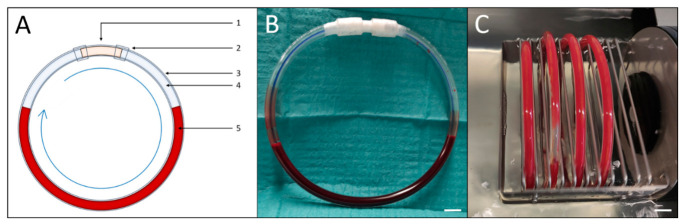
Construction of loop containing sample prior to Chandler Loop testing. (**A**): schematic outline of loop construction; 1: Tube containing implant, 2: Connectors, 3: Tube, 4: Air, 5: 7 mL whole human blood; The blue arrow marks the direction of the rotation (**B**): noDOP-tube containing a sample graft and human whole-blood before the test run; (**C**): top view of sample loops during test run. Scale bar: 20 mm.

The in vitro testing was performed in eight independent test runs with four tubes cultivated in parallel in each run: one containing the fibrin-based graft, one containing the heparin-coated fibrin graft (Fibrin Graft+), one tube containing the VascuGraft, and one control tube without a sample. Following the preparation and insertion of the fibrin-based grafts with and without heparin coating as well as the synthetic prosthesis, each of the three samples was integrated into a loop by connecting the ends of the specimen tubes to those of the testing tube, creating a loop with a total tube length of 53.5 cm and a corresponding radius of 8.5 cm (Figure 2A,B). For tubing, phthalate-free soft-PVC-based tubes (noDOP^®^, Raumedic AG, Helmbrechts, Germany) with an inner diameter of 6.35 mm and a wall thickness of 1.5875 mm were used. Serving as a control, a fourth tube was prepared using only the tube without containing any samples. All four tubes were then filled with 7 mL of whole blood supplemented with sodium citrate (10.9 mmol/L) before installation into the Chandler Loop System^®^ (Ebo Kunze Industriedesign, Neuffen, Germany) (Figure 2C). Blood was drawn from healthy donors (*n* = 8) between 20 and 35 years of age with an equal number of male and female participants after obtaining written informed consent. This study was conducted with the approval of the Ethics Committee of Hannover Medical School.

The loops were circulated at a rotation rate of 30 rpm in a water bath facilitating a consistently controlled temperature of 37 °C for four hours (Supplementary Video S1). This time period was chosen to ensure sufficient contact time between the prostheses and the blood while limiting effects associated with impaired leukocyte and platelet function during extended ex vivo whole-blood incubation. After ending the test run, the samples were carefully removed from the loops and stored in a fixation solution containing 1.5% Paraformaldehyde, 1.5% Glutaraldehyde and 150 mM HEPES buffer at pH 7.35. Both, the blood from the four test tubes per run, and a blood sample from each donor prior to the test run, which was used as a baseline reference, were collected.

### 2.3. Analysis

The total cell count of erythrocytes, thrombocytes, and leukocytes, as well as the hematocrit and hemoglobin concentration, were investigated. For this, whole blood samples were analyzed in cooperation with the Institute for Clinical Chemistry at Hannover Medical School using the standard technique used for clinical blood count determination. Additionally, plasma samples of each group were obtained by centrifugation of the blood probes at 2000× *g*. The plasma collected as supernatant was used for subsequent ELISA analysis. ELISA-analysis was performed to determine the concentrations of mediators relevant for the plasmatic coagulation cascade, the complement-mediated coagulation, and thrombocyte-mediated coagulation. As an indicator for activation of the plasmatic coagulation cascade, the thrombin–antithrombin complex (TAT), representative for plasmatic thrombin activation, was quantified (Enygnost TAT Kit micro, Siemens, Munich, Germany). Additionally, sC5b-9 was measured as a marker for terminal activation of the complement-system (MicroVue sC5b-9 Plus EIA, Quidel, San Diego, CA, USA), and P-Selectin was analyzed as an indicator for thrombocyte activation (Human P-Selectin/CD62P Immunoassay Quantikine^®^, RD Systems, Inc., Minneapolis, MN, USA). To exclude a possible interfering influence of the tubing, the absolute values for each group were ratioed to the sample-free control tube in each run.

For scanning electron microscopic assessment of the luminal surface of the grafts, two 5–6 mm long segments were cut from the center part of each specimen following each test run. The segments were then prepared for scanning electron microscopy (SEM) using Zeiss Crossbeam 540-47-80 microscope (Carl Zeiss, Oberkochen, Germany) (Institute of Functional and Applied Anatomy, Hannover Medical School). Samples were analyzed in 1.500 × magnification capturing plane views of the luminal surface.

### 2.4. Statistics

Statistical analysis was performed using IBM SPSS Statistics 29 (IBM, Armok, NY, USA). The data was analyzed as a paired analysis for each test run (*n* = 8), which was conducted in parallel for the respective groups using the whole-blood sample from a single donor respectively. For comparisons between groups, One-way ANOVA was performed. Values are given as mean ± standard deviation. Results were considered significant at *p* < 0.05 and marked with an asterisk in the corresponding graphs.

## 3. Results

### 3.1. Hemocompatibility Analysis

Irrespective of the specimen, there were no incidents of thrombotic occlusions detected throughout the experiments. The loop as well as the incorporated samples remained intact and in place for the time of the Chandler Loop test run.

**Figure 3:**
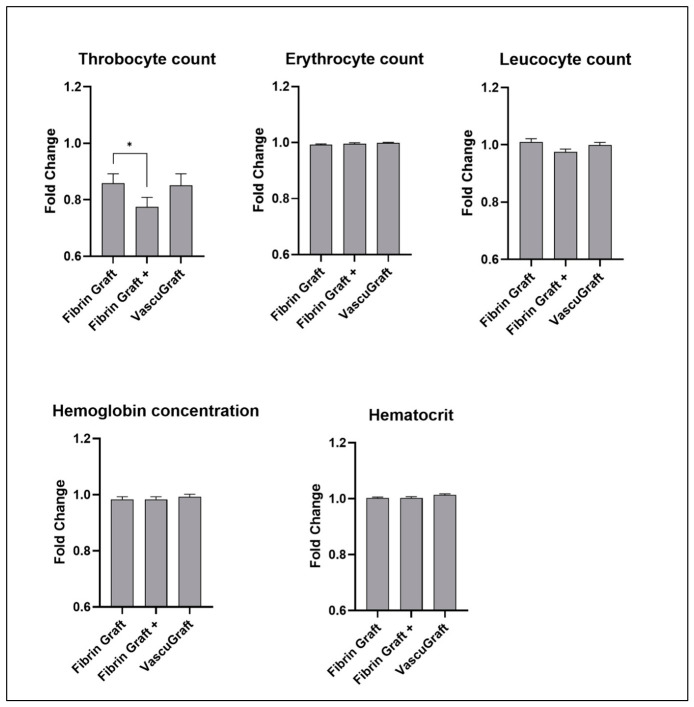
Cell count analysis. Relative changes in the thrombocyte, erythrocyte, and leucocyte cell count, hemoglobin concentration, and hematocrit levels after 4 h of incubation in the Chandler Loop test tubes compared to the pre-test baseline values. Mean values and standard deviation are shown. * *p* < 0.05.

In order to examine possible cell adhesion and hemolysis, the number of thrombocytes, erythrocytes, leukocytes as well as the hemoglobin and hematocrit levels after the test run were determined and compared to the baseline values acquired prior to the test run. Thrombocyte consumption was present in all groups with lower thrombocyte counts compared to the baseline values. Here, the fibrin-based grafts without heparin coating and the synthetic heparin-coated VascuGraft showed a similar decrease in the thrombocyte counts with 85.8% ± 8.9% of the baseline value for the fibrin grafts and 85.0% ± 11.1% for the VascuGrafts respectively (*p* = 0.956, Figure 3). Compared to that, the thrombocyte loss was distinctively higher in the group of the heparin-coated fibrin grafts with only 77.5% remaining after the test run, (*p* = 0.046). No significant decreases in the cell count could be observed in the erythrocyte and leucocyte count in every group with ratios near 1.0 compared to the baseline values (Figure 3). Similarly, no significant changes in the hemoglobin levels and hematocrit were observed.

Fluorescence microscopic analysis verified the presence of heparin on the luminal surface of the heparin-coated fibrin grafts and heparinized VascuGrafts (Supplementary Figure S1).

**Figure 4:**
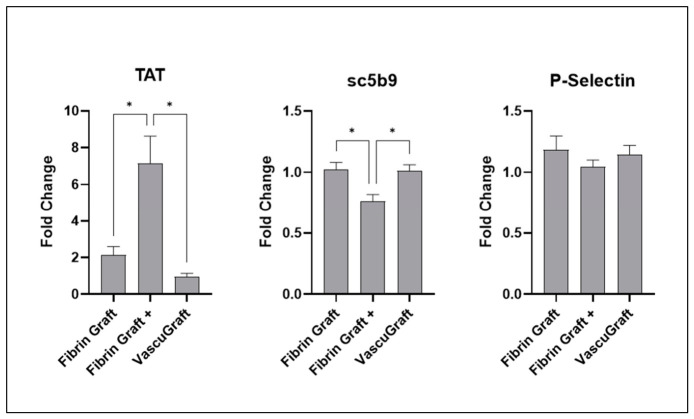
ELISA analysis. Relative changes in the concentrations of thrombin–antithrombin complex (TAT), Complement sC5b-9, and P-Selectin after 4 h of incubation in the Chandler Loop test tubes compared to the pre-test baseline values. Mean values and standard deviation are shown. * *p* < 0.05.

ELISA analysis of the thrombin–antithrombin complex revealed a nearly two-fold increase in plasmatic TAT-levels in fibrin grafts after incubation in the Chandler Loop compared to the baseline-values (Figure 4). TAT-levels were even higher in the heparinized “Fibrin+” grafts, while no significant increase in the thrombin–antithrombin complex levels was present in the VascuGrafts. Concerning complement activation, no relevant increase in sC5b-9 levels were observed in the fibrin grafts, while sC5b-9 levels were even decreased below the baseline in the heparin-coated fibrin grafts. P-Selectin levels did not differ significantly from the baseline values in each test group and no significant differences were found among the groups.

### 3.2. Scanning Electron Microscopic Assessment

**Figure 5:**
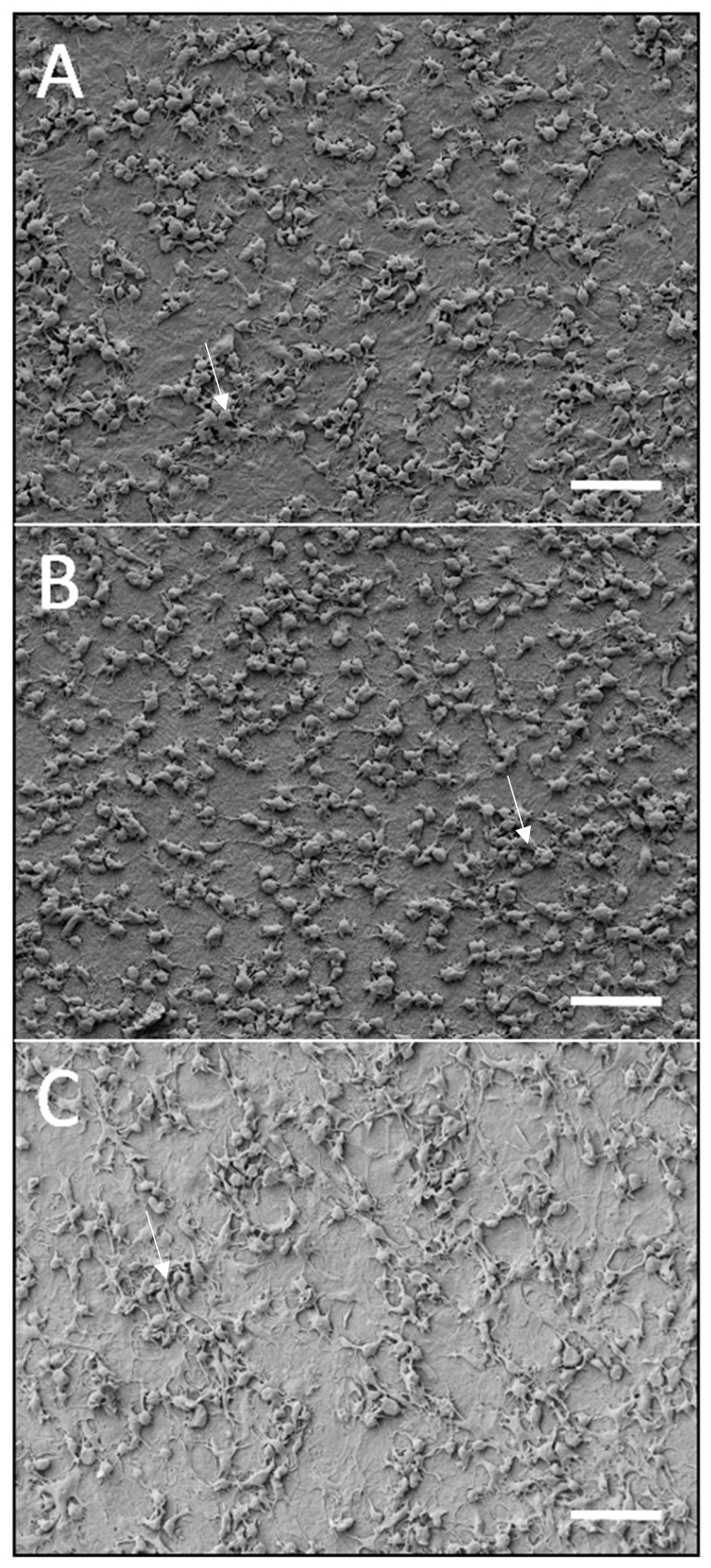
Scanning electron microscopy of luminal surface of the grafts after 4 h incubation in the Chandler Loop system. (**A**): fibrin graft; (**B**): fibrin graft with heparin coating (Fibrin+); (**C**): synthetic vascular graft with heparin coating (VascuGraft). Arrows show exemplary activated thrombocyte aggregates. Magnification: 1500×, Scale bar: 10 µm.

To assess possible adhesion of thrombocytes, leucocytes, or erythrocytes, as well as potential wall-adherent thrombus formation on the luminal surface of the samples, scanning electron microscopy was performed. Here, adhesion of thrombocytes on the luminal surface could be observed in all three groups (Figure 5). With respect to cell morphology, adhesive thrombocytes showed pseudopodia formation in all samples indicating an activated status of the thrombocytes. Platelets were distributed homogenously in all samples without morphological evidence of specific predisposing areas within the luminal surface of the grafts. In contrast to that, no other attached cells or clot formation were observed on the graft surface, with exception of few sparse and isolated erythrocytes.

## 4. Discussion

The main findings of this study can be summarized as follows: (1) The Chandler Loop model proved to be a suitable test method for the in vitro evaluation of small-diameter bioartificial and synthetic vascular grafts with sufficient study inherent reliability. (2) Small-diameter fibrin-based vascular grafts showed comparable hemocompatibility in the in vitro short-term Chandler Loop model compared to heparin-coated synthetic grafts.

### 4.1. Hemocompatibility Assessment and Application of the Chandler Loop Model

The Chandler Loop model proved to be a suitable system for the in vitro assessment of hemocompatibility in our small-diameter vascular grafts in accordance with DIN ISO 10993-4 guidelines for the evaluation of blood-contacting medical devices.

As hemocompatibility remains a vital constraint for the clinical translation of blood-contacting materials, it is essential to thoroughly investigate potential interactions and effects under defined and physiologically relevant test conditions already at an early stage in the development of biomimetic materials [15,17,18]. In recent years, the Chandler Loop has gained recognition as a reliable and well-established dynamic in vitro model for hemocompatibility testing in accordance with ISO 10993-4, as demonstrated by its successful application in numerous approaches [19,20,21]. For instance, applying a similar set of test categories as performed here, Sinn et al. investigated the feasibility of the Chandler Loop model for hemocompatibility testing of different stent types [22]. In line with these findings, the homogeneous distribution of platelets on the luminal surface of the probes seen in the electron microscopic assessment in our study suggests stable flow conditions within the probes in the Chandler Loop model. However, as illustrated by Van Oeveren et al., the Chandler Loop (as any current test model) exhibits limitations despite its widespread use. The main drawback of this model is the air-liquid interface that can introduce artifacts through unnatural shear forces, potentially leading to protein denaturation and activation of blood components [23]. Furthermore, ex vivo whole blood culture is only suitable for short-term incubation periods. Thus, the Chandler Loop setup is not the only approach in search for an in vitro model for hemocompatibility testing. Other common test methods include a roller pump-based perfusion system for thrombogenicity testing. For example, Jamiolkowski et al. used a roller pump model to assess the thrombogenicity of different materials using human blood based on thrombus coverage and platelet count reduction [24]. While the authors were able to effectively differentiate the thrombogenicity profile using this test system, the roller pump remains inherently limited by its tendency to induce platelet reduction and hemolysis due to the exposure to severe mechanical stress [25]. In contrast, the modified Chandler Loop’s balanced rotational movement causes less shear-induced damage or trauma, rendering it more suitable for evaluating sensitive blood parameters. In overview of the currently available literature, and in accordance with our study inherent findings, the Chandler Loop model may currently represent the most appropriate setup for reproducible, physiologically relevant hemocompatibility testing in this specific application. Thus, this model was chosen for the comparative in vitro analysis in our study. In combination with standard cell-count analyses, this setup also facilitated quantitative assessment of cell consumption and adhesion.

### 4.2. Hemocompatibility of Fibrin as a Matrix Material for Bioartificial Vascular Grafts

In search for an ideal scaffold material for tissue engineering approaches, fibrin has gained increasing attention in recent years [6,26,27]. Particularly in the context of the challenging requirements in vascular tissue engineering, where the scaffolds must withstand both mechanical stress and direct blood contact, fibrin-based materials have been proven beneficial not only due to their mechanical performance but also by their biological suitability. In previous works, fibrin-based constructs with and without endothelial cell linings were tested under physiological pressure- and flow conditions in hemodynamic simulation systems. These investigations proved the general suitability of fibrin as a matrix material for vascular grafts that withstand physiological biomechanical stressors [13,28,29,30]. Further works have focused on cellularization capacity with a special emphasis on luminal endothelialization [8,9,31]. In addition to these factors, that have been addressed previously, hemocompatibility remains a key factor for clinical applicability of blood-contacting implant materials, especially for small-diameter vascular prosthesis. Thus, in the present study, we focused on evaluating the hemocompatibility concerning fibrin-based, small-caliber vascular grafts. To allow off-the-shelf- availability with respect to future translational applications of the fibrin grafts, as well as to facilitate comparison to synthetic graft materials, the fibrin grafts were tested without luminal endothelialization. Nonetheless, previous studies have shown the general suitability of fibrin-based vessels for luminal endothelial cell seeding under static and dynamic conditions [12,13]. The results in this early-stage study showed that fibrin-based vascular grafts exhibited hemocompatibility properties comparable to those of the heparin-coated ePTFE grafts even without heparin coating of the fibrin-based prosthesis. While platelet activation and adhesion could be observed in the fibrin vessels as well, this did not differ significantly from the heparin-coated synthetic graft material (Figure 3 and Figure 4). Concerning activation of the plasmatic coagulation system with the thrombin–antithrombin complex (TAT) serving as a surrogate parameter in our study, fibrin-based grafts showed a tendency towards higher TAT levels compared to the synthetic heparin-coated VascuGrafts. This effect was even more pronounced in the heparin-coated fibrin vessels. However, this observation may be explained by the residual soluble thrombin remaining in the matrix, which was used during the polymerization process of the fibrin matrix and led to a relatively high increase in TAT concentration in the small blood volume of the Chandler Loop. While the tendency towards higher activation of the plasmatic coagulation in fibrin-based grafts did not reach statistical significance compared to the heparinized synthetic grafts, and the clinical implications of residual soluble thrombin in the larger blood volume of the human circulatory system remain unclear, possible measures in future approaches could include more intense washing and rinsing of the grafts after polymerization to eliminate residual thrombin. There were no significant changes in the cell count of leucocytes indicating no relevant leucocyte adhesion and/or transmigration in any of the probes. Accordingly, scanning electron microscopy showed no adhesive leucocytes on the graft surfaces. Furthermore, no significant hemolysis was present as demonstrated by stable hemoglobin and hematocrit levels. Likewise, no significant complement activation as indicated by sC5b-9 was initiated by both, the fibrin-based grafts and synthetic heparin-coated grafts. Thus, in summary, fibrin-based grafts showed acceptable hemocompatibility with regard to the parameters tested in this study. In this initial investigation, these results were comparable to those of the current gold standard for prosthetic vascular replacement, represented by the heparin-coated VascuGraft.

These first findings line up with other studies investigating the material properties of fibrin as reviewed by Rojas-Murillo et al. [32]. As a further example, Kaplan et al. demonstrated low-thrombogenicity and favorable endothelialization potential of fibrin- and fibrin-heparin coatings in vitro, highlighting the inherent blood-contact safety profile [19]. However, while these previous studies have demonstrated promising hemocompatibility profiles of fibrin-based materials, most of these investigations were often limited to combinations of materials or explored simplified experimental setups such as static assays or surface coatings, thus not reflecting the complex requirements of small-caliber vascular grafts, where three-dimensional structure, flow dynamics, and material integration play a critical role. The present study addressed this by evaluating fibrin-based vascular constructs under physiologically relevant flow conditions using human blood. This serves as a first insight into the hemocompatibility of fibrin- based small-diameter vessels, supporting the suitability of fibrin as a scaffold material in vascular tissue engineering.

Since vascular graft performance strongly depends on biomechanical properties, it is pivotal to consider the mechanical characteristics of fibrin-based small-caliber grafts when interpreting hemocompatibility results. Small-diameter vascular grafts fabricated in the same manner as the probes used in the present study exhibited a maximum burst pressure of around 200 mmHg, an absolute uniaxial tensile strength of up to 1.5 N and yielded physiological elastic behavior comparable to native arteries in previous studies [8,9].

As alternatives to fibrin, a variety of biomaterials and synthetic scaffold materials have been extensively investigated for their suitability in vascular tissue engineering approaches. One of the most widely used biomaterials is collagen, valued for its cytocompatibility and its inherent ability to support endothelial adhesion and migration [33,34]. However, despite these advantages, collagen is known to activate platelet aggregation, as it binds directly to platelet surface receptors, causing thrombus formation [35,36]. In contrast, the fibrin-based bioartificial grafts showed only low reduction in platelet count and no significant increase in the thrombocyte activation marker P-Selectin. The current gold standard material for the prosthetic replacement of small diameter vessels is expanded polytetrafluoroethylene (ePTFE), which is widely used in vascular surgery. ePTFE offers mechanical stability and a low acute immune response [37]. Moreover, in vitro studies showed distinctively lower complement activation by ePTFE compared to polyethylene terephthalate (Dacron^®^) [38]. These findings are consistent with our observations, where no significant increases in the level of complement sC5b-9 was observed. However, ePTFE lacks bioactive properties and does not support cell ingrowth and microvascular integration, resulting in a risk for biofilm formation and chronic graft infection [39,40]. Moreover, lacking endothelial coverage and insufficient compliance properties is particularly problematic in small-caliber grafts, often resulting in long-term occlusion [41].

While these materials such as collagen or ePTFE offer many beneficial characteristics, fibrin combines hemocompatibility with biological activity and functional adaptability. The tendency towards low thrombogenicity, low complement activation and hemolytic potential comparable to that of the ePTFE grafts provide further evidence for a possible advantageous blood-contact safety profile of fibrin-based vascular scaffolds even in small-diameter grafts.

### 4.3. Heparin-Coating of Fibrin-Based Vascular Grafts

Heparin-coating has been a widely used strategy to improve the bio- and hemocompatibility of blood-contacting implant materials for many years, as it inhibits thrombin generation, reduces platelet adhesion, and may also attenuate complement activation in certain contexts [42,43]. The rationale for the use of thrombin as a mediator for heparin-coating of fibrin-based grafts is based on the transient formation of a ternary heparin-thrombin-fibrin complex based on binary interactions between heparin and thrombin, thrombin and fibrin, and heparin and fibrin, as previously reported by Liaw et al. [44]. Here, we compared heparin-coated fibrin-grafts with non-coated fibrin grafts to evaluate the impact of an additional heparin coating and whether it may further enhance hemocompatibility. In accordance with most reports of the beneficial use of heparin, it could be observed that heparin-coated specimen activated the complement system to a lesser degree than uncoated fibrin grafts as indicated by lower sC5b-9 levels. In contrast, an adverse effect observed in heparin-coated fibrin grafts with respect to the TAT complex-formation.

While the use of heparin undeniably offers many benefits, not only in regard of the widespread experience in its application but also due to the possibilities to improve short-term patency of blood-contacting devices, heparin coatings are limited in their long-term stability: mechanical stress, blood flow, and enzymatic processes can degrade or remove the coating over time, which may be one of the factors leading to the limited long-term patency rates observed even in the current gold-standard heparin-coated ePTFE-grafts [45,46]. Furthermore, heparin exerts only passive effects by preventing thrombus formation, but it does not promote active healing or integration of the graft into the surrounding tissue, which is a key factor for successful long-term effects of biomimetic implants.

In our study, heparin-coating of fibrin-based small-diameter vascular grafts arguably did not further increase the in vitro hemocompatibility of the grafts. It should, however, be noted that the uncoated fibrin grafts showed hemocompatibility results comparable to those obtained for heparin-coated synthetic grafts.

### 4.4. Limitations

It should be considered that this ex vivo setup does not fully reflect the physiological biomechanical environment of the human vascular system including factors such as pulsatile flow, cyclic stretch, and shear stress gradients, and the Chandler Loop analysis is limited to short-term effects. SEM analysis was limited to qualitative assessment only, while cell-count analysis facilitated quantification of the cell adhesion during the incubation period. Potential long-term effects such as chronic inflammation, surface degradation or endothelial regeneration remain unaddressed at this point, which is why further investigations, especially focusing on long-term in vivo applications, are needed. Additionally, it should be considered that residual thrombin used in the protocol for heparin coating may have had a confounding effect on the partially increased thrombogenicity observed in the heparin-coated grafts, particularly with respect to the elevated TAT-levels.

The test parameters used here were chosen on basis of the ISO 10993-4 standard and constitute only a selection of available tests and cannot fully capture the entire spectrum of blood–material interactions. Moreover, this study represents an early-stage investigation and thus does not prove translational readiness.

## 5. Conclusions

Our findings demonstrate that small-diameter bioartificial fibrin-based vascular grafts exhibit hemocompatibility comparable to the current clinical gold-standard of heparin-coated ePTFE. Heparin coating might not be necessary in fibrin-based grafts, since the bioartificial grafts exhibited hemocompatibility characteristics in the range of heparin-coated synthetic grafts even without coating. In summary, the present early-stage findings support fibrin as a promising scaffold material for small-diameter vascular tissue engineering. While these results are encouraging, further studies must investigate the extent to which these properties also hold true in long-term in vivo trials.

## Data Availability

The original contributions presented in this study are included in the article/Supplementary Materials. Further inquiries can be directed to the corresponding authors.

## Supplementary Materials

The following supporting information can be downloaded at: https://www.mdpi.com/article/10.3390/jfb17060303/s1, Figure S1: Heparin coating. Immunofluorescence microscopy for epitope 10E4. (**A**): cross section of the wall of a heparin-coated fibrin graft, (**B**): cross section of a fibrin graft without heparin coating (negative control), (**C**): luminal view of the heparin-coated VascuGraft (positive control). The dotted line marks the luminal border of the graft wall in the cross-section images. Scale bar = 100 µm. Video S1: Chandler loop working principle. The rotating Chandler loop system during a test run with four test tubes is shown.

## References

[1] Bettencourt P.J., GBD 2021 Demographics Collaborators (2024). Global age-sex-specific mortality, life expectancy, and population estimates in 204 countries and territories and 811 subnational locations, 1950–2021, and the impact of the COVID-19 pandemic: A comprehensive demographic analysis for the Global Burden of Disease Study 2021. Lancet.

[2] Abbott W.M., Green R.M., Matsumoto T., Wheeler J.R., Miller N., Veith F.J., Suggs W.D., Hollier L., Money S., Garrett H. (1997). Prosthetic above-knee femoropopliteal bypass grafting: Results of a multicenter randomized prospective trial. Above-Knee Femoropopliteal Study Group. J. Vasc. Surg..

[3] Green R.M., Abbott W.M., Matsumoto T., Wheeler J.R., Miller N., Veith F.J., Money S., Garrett H.E. (2000). Prosthetic above-knee femoropopliteal bypass grafting: Five-year results of a randomized trial. J. Vasc. Surg..

[4] Jackson M.R., Belott T.P., Dickason T., Kaiser W.J., Modrall J., Valentine R., Clagett G. (2000). The consequences of a failed femoropopliteal bypass grafting: Comparison of saphenous vein and PTFE grafts. J. Vasc. Surg..

[5] Yamamoto Y., Uchiyama H., Oonuki M., Tsukuda K., Kazama A., Wada Y., Kikuchi T., Nishizawa M., Kudo T. (2024). Long-term outcomes of below-the-knee bypass surgery using heparin-bonded expanded polytetrafluoroethylene grafts. Surg. Today.

[6] Shaikh F.M., Callanan A., Kavanagh E.G., Burke P.E., Grace P.A., McGloughlin T.M. (2008). Fibrin: A natural biodegradable scaffold in vascular tissue engineering. Cells Tissues Organs.

[7] Lau S. (2017). Strategies for the Generation of Fully Autologous Tissue-Engineered Fibrin-Based Vascular Grafts Resembling Three-Layered Natural Arteries.

[8] Helms F., Haverich A., Böer U., Wilhelmi M. (2021). Transluminal compression increases mechanical stability, stiffness and endothelialization capacity of fibrin-based bioartificial blood vessels. J. Mech. Behav. Biomed. Mater..

[9] Aper T., Wilhelmi M., Gebhardt C., Hoeffler K., Benecke N., Hilfiker A., Haverich A. (2016). Novel method for the generation of tissue-engineered vascular grafts based on a highly compacted fibrin matrix. Acta Biomater..

[10] Aper T., Wilhelmi M., Boer U., Lau S., Benecke N., Hilfiker A., Haverich A. (2018). Dehydration improves biomechanical strength of bioartificial vascular graft material and allows its long-term storage. Innov. Surg. Sci..

[11] Wilhelmi M., Jockenhoevel S., Mela P. (2014). Bioartificial fabrication of regenerating blood vessel substitutes: Requirements and current strategies. Biomed. Tech..

[12] Helms F., Lau S., Aper T., Zippusch S., Klingenberg M., Haverich A., Wilhelmi M., Böer U. (2021). A 3-Layered Bioartificial Blood Vessel with Physiological Wall Architecture Generated by Mechanical Stimulation. Ann. Biomed. Eng..

[13] Glomb C., Wilhelmi M., Strauß S., Zippusch S., Klingenberg M., Aper T., Vogt P.M., Ruhparwar A., Helms F. (2024). Fabrication and biomechanical characterization of a spider silk reinforced fibrin-based vascular prosthesis. J. Mech. Behav. Biomed. Mater..

[14] Mueller M., Krolitzki B., Glasmacher B. (2012). Dynamic in vitro hemocompatibility testing—Improving the signal to noise ratio. Biomed. Eng..

[15] Weber M., Steinle H., Golombek S., Hann L., Schlensak C., Wendel H.P., Avci-Adali M. (2018). Blood-Contacting Biomaterials: In Vitro Evaluation of the Hemocompatibility. Front. Bioeng. Biotechnol..

[16] Chandler A.B. (1958). In vitro thrombotic coagulation of the blood; a method for producing a thrombus. Lab. Investig..

[17] Blok S.L.J., Engels G.E., van Oeveren W. (2016). In vitro hemocompatibility testing: The importance of fresh blood. Biointerphases.

[18] Braune S., Grunze M., Straub A., Jung F. (2013). Are there sufficient standards for the in vitro hemocompatibility testing of biomaterials?. Biointerphases.

[19] Kaplan O., Hierlemann T., Krajewski S., Kurz J., Nevoralová M., Houska M., Riedel T., Riedelová Z., Zárubová J., Wendel H.P. (2017). Low-thrombogenic fibrin-heparin coating promotes in vitro endothelialization. J. Biomed. Mater. Res. A.

[20] Avci-Adali M., Grözinger G., Cabane V., Schreve M., Wendel H.P. (2023). Improving Bioactive Characteristics of Small Diameter Polytetrafluoroethylene Stent Grafts by Electrospinning: A Comparative Hemocompatibility Study. Bioengineering.

[21] Nalezinková M. (2020). In vitro hemocompatibility testing of medical devices. Thromb. Res..

[22] Sinn S., Scheuermann T., Deichelbohrer S., Ziemer G., Wendel H.P. (2011). A novel in vitro model for preclinical testing of the hemocompatibility of intravascular stents according to ISO 10993-4. J. Mater. Sci. Mater. Med..

[23] van Oeveren W., Tielliu I.F., de Hart J. (2012). Comparison of modified chandler, roller pump, and ball valve circulation models for in vitro testing in high blood flow conditions: Application in thrombogenicity testing of different materials for vascular applications. Int. J. Biomater..

[24] Jamiolkowski M.A., Hartung M.C., Malinauskas R.A., Lu Q. (2020). An In Vitro Blood Flow Loop System for Evaluating the Thrombogenicity of Medical Devices and Biomaterials. ASAIO J..

[25] Furugaki T., Shigeta O., Kozuma Y., Tsukada T., Nakajima T., Sakamoto H., Mathis B.J., Hiramatsu Y., Suzuki Y. (2021). The effect of roller head pump on platelet deterioration during the simulated extracorporeal circulation. J. Artif. Organs.

[26] Lang Z., Chen T., Zhu S., Wu X., Wu Y., Miao X., Wang Q., Zhao L., Zhu Z., Xu R.X. (2024). Construction of vascular grafts based on tissue-engineered scaffolds. Mater. Today Bio..

[27] Li S., Dan X., Chen H., Li T., Liu B., Ju Y., Li Y., Lei L., Fan X. (2024). Developing fibrin-based biomaterials/scaffolds in tissue engineering. Bioact. Mater..

[28] Käding C.D., Glomb C.-S., Stadler P., Becker I., Klingenberg M., Höffler H.-K., Pflaum M., Ruhparwar A., Wilhelmi M., Helms F. (2026). Generation of Fibrin-Based Aortic Vessels with Layer-Specific Cell Architecture Under Pulsatile Perfusion in a Clinical Organ Care System. Ann. Biomed. Eng..

[29] Helms F., Zippusch S., Aper T., Kalies S., Heisterkamp A., Haverich A., Böer U., Wilhelmi M. (2022). Mechanical stimulation induces vasa vasorum capillary alignment in a fibrin-based tunica adventitia. Tissue Eng. Part A.

[30] Bobylev D., Wilhelmi M., Lau S., Klingenberg M., Mlinaric M., Petená E., Helms F., Hassel T., Haverich A., Horke A. (2021). Pressure-compacted and spider silk-reinforced fibrin demonstrates sufficient biomechanical stability as cardiac patch in vitro. J. Biomater. Appl..

[31] Helms F., Haverich A., Wilhelmi M., Böer U. (2021). Establishment of a Modular Hemodynamic Simulator for Accurate In Vitro Simulation of Physiological and Pathological Pressure Waveforms in Native and Bioartificial Blood Vessels. Cardiovasc. Eng. Technol..

[32] Rojas-Murillo J.A., Simental-Mendía M.A., Moncada-Saucedo N.K., Delgado-Gonzalez P., Islas J.F., Roacho-Pérez J.A., Garza-Treviño E.N. (2022). Physical, Mechanical, and Biological Properties of Fibrin Scaffolds for Cartilage Repair. Int. J. Mol. Sci..

[33] Simionescu D.T., Lu Q., Song Y., Lee J., Rosenbalm T.N., Kelley C., Vyavahare N.R. (2006). Biocompatibility and remodeling potential of pure arterial elastin and collagen scaffolds. Biomaterials.

[34] Post A., Wang E., Cosgriff-Hernandez E. (2019). A Review of Integrin-Mediated Endothelial Cell Phenotype in the Design of Cardiovascular Devices. Ann. Biomed. Eng..

[35] Harada K., Wenlong W., Shinozawa T. (2024). Physiological platelet aggregation assay to mitigate drug-induced thrombocytopenia using a microphysiological system. Sci. Rep..

[36] Induruwa I., Moroi M., Bonna A., Malcor J.-D., Howes J.-M., Warburton E.A., Farndale R., Jung S. (2018). Platelet collagen receptor Glycoprotein VI-dimer recognizes fibrinogen and fibrin through their D-domains, contributing to platelet adhesion and activation during thrombus formation. J. Thromb. Haemost..

[37] Roina Y., Auber F., Hocquet D., Herlem G. (2022). ePTFE-based biomedical devices: An overview of surgical efficiency. J. Biomed. Mater. Res. B Appl. Biomater..

[38] Shepard A.D., Gelfand J.A., Callow A.D., O’Donnell T.F. (1984). Complement activation by synthetic vascular prostheses. J. Vasc. Surg..

[39] Kirkton R.D., Prichard H.L., Santiago-Maysonet M., Niklason L.E., Lawson J.H., Dahl S.L.M. (2018). Susceptibility of ePTFE vascular grafts and bioengineered human acellular vessels to infection. J. Surg. Res..

[40] Wang J., Blalock S.K.F., Levitan G.S., Prichard H.L., Niklason L.E., Kirkton R.D. (2023). Biological mechanisms of infection resistance in tissue engineered blood vessels compared to synthetic expanded polytetrafluoroethylene grafts. JVS Vasc. Sci..

[41] Fayon A., Menu P., El Omar R. (2021). Cellularized small-caliber tissue-engineered vascular grafts: Looking for the ultimate gold standard. npj Regen. Med..

[42] Biran R., Pond D. (2017). Heparin coatings for improving blood compatibility of medical devices. Adv. Drug Deliv. Rev..

[43] Wendel H.P., Ziemer G. (1999). Coating-techniques to improve the hemocompatibility of artificial devices used for extracorporeal circulation. Eur. J. Cardiothorac. Surg..

[44] Liaw P.C., Becker D.L., Stafford A.R., Fredenburgh J.C., Weitz J.I. (2001). Molecular basis for the susceptibility of fibrin-bound thrombin to inactivation by heparin cofactor ii in the presence of dermatan sulfate but not heparin. J. Biol. Chem..

[45] Jarad N.A., Chami A., Weitz J.I., Didar T.F. (2024). Advancements in surface modification strategies of vascular grafts to improve biocompatibility and tissue integration. Explor. BioMat-X.

[46] Gore S., Andersson J., Biran R., Underwood C., Riesenfeld J. (2014). Heparin surfaces: Impact of immobilization chemistry on hemocompatibility and protein adsorption. J. Biomed. Mater. Res. B Appl. Biomater..

