# Supplementary figures and images for: Is Fibrin a Suitable Matrix for Small-Diameter Bioartificial Vascular Grafts? An In Vitro Short-Term Hemocompatibility Study
Source: J Funct Biomater. 2026 Jun 18;17(6):303. doi: 10.3390/jfb17060303 (PMC13301373; doi:10.3390/jfb17060303)

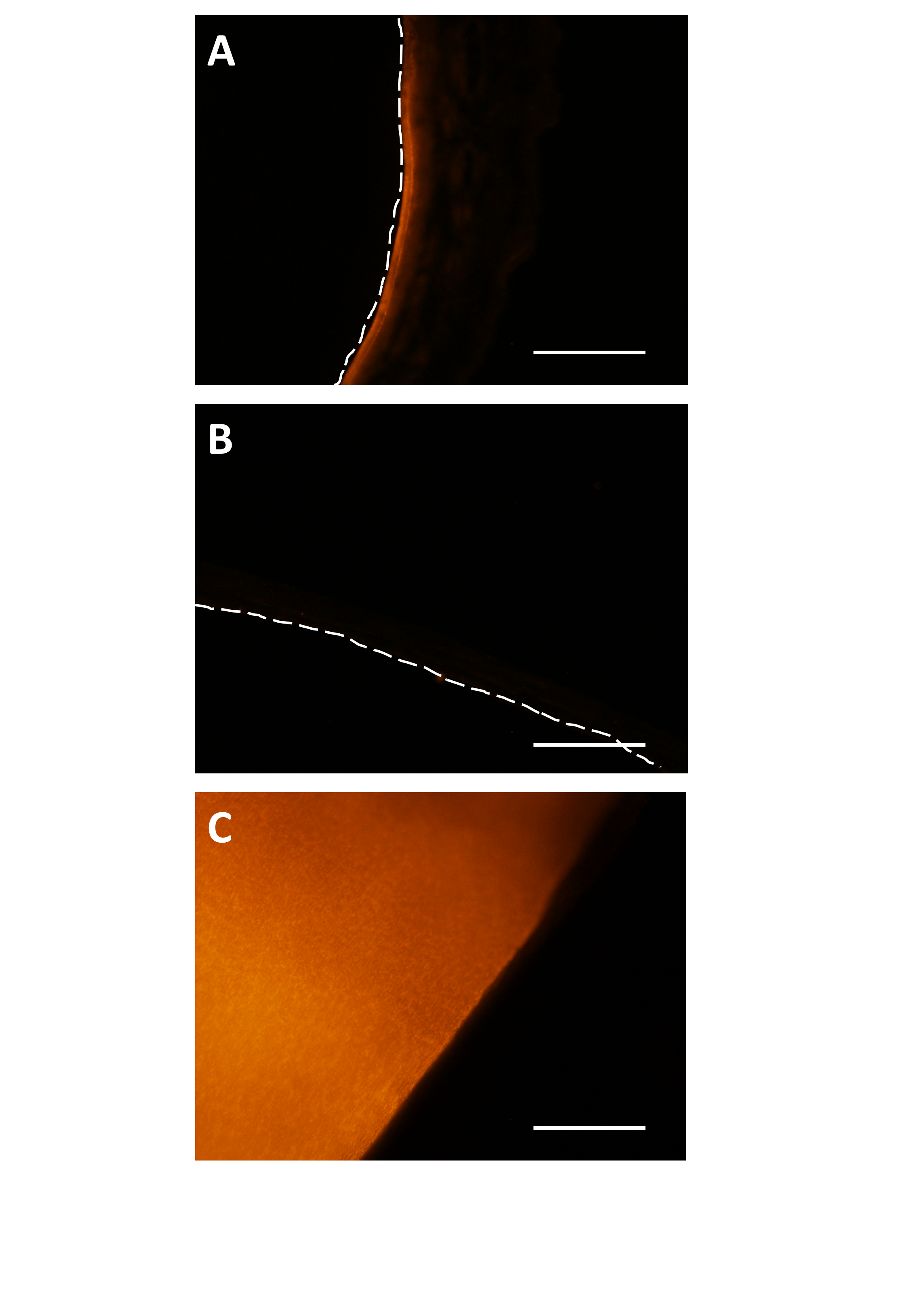

Supplement: Supplementary file 1 [file jfb-17-00303-s001.zip › Supplemental Figure S1.tif]
